# *Cyclovirus*-Related DNA in Bovine Feces from Northern Portugal: Molecular Detection and Phylogenetic Characterization

**DOI:** 10.3390/microorganisms14102374

**Published:** 2026-10-16

**Authors:** Ricardo J. Figueiredo, Sara Gomes-Gonçalves, Guilherme Moreira, Kimberly Huettich, Salome Klaus, Maja Corley, Katharina Prusa, Beatriz Oliveira Alcobia, João R. Mesquita

**Affiliations:** 1 School of Medicine and Biomedical Sciences (ICBAS), Universidade do Porto (UP), 4050-313 Porto, Portugal; up202206087@up.pt (R.J.F.); up201904503@up.pt (B.O.A.); 2 Landesbetrieb Hessisches Landeslabor (LHL), Schubertstraße 60, Haus 13, 35392 Gießen, Germany; kimberlyhuettich17@gmail.com (K.H.);; 3 LAQV, REQUIMTE, Department of Chemistry and Biochemistry, Faculty of Sciences, University of Porto, 4099-007 Porto, Portugal

**Keywords:** *Cyclovirus*, *Circoviridae*, CRESS-DNA viruses, cattle, replication-associated protein (Rep), molecular epidemiology, host association

## Abstract

Cycloviruses are circular Rep-encoding single-stranded DNA (CRESS-DNA) viruses detected in a wide range of biological and environmental samples, although the natural hosts and biological significance of many members of the genus remain poorly understood. Information regarding their occurrence in cattle, particularly in fecal material, remains limited. In this study, 100 bovine fecal samples collected from 21 farms in northern Portugal were screened for *circovirus*- and *cyclovirus*-related DNA using a nested PCR assay targeting a conserved region of the replication-associated protein (*rep*) gene. Amplicons were characterized by bidirectional Sanger sequencing and maximum-likelihood phylogenetic analysis. *Cyclovirus*-related DNA was detected in 14/100 samples, corresponding to a molecular occurrence of 14.0% (cluster-bootstrap 95% CI: 5.6–26.5%), with positive animals originating from 7/21 farms. The obtained partial *rep* sequences showed 94.8–100% nucleotide identity with *Crocidura russula* feces-associated *Cyclovirus* MAVG5 (PQ576920), and five sequences were identical to the corresponding homologous region. Phylogenetic analysis grouped all bovine-derived sequences with MAVG5 within a broader lineage containing Human-associated *Cyclovirus* 6. In an exploratory GEE analysis accounting for within-farm clustering, no statistically significant association was observed between cyclovirus detection and diarrhea (GEE: OR = 0.76, 95% CI: 0.31–1.86; *p* = 0.549). These findings expand current knowledge of *Cyclovirus*-related DNA in livestock-associated fecal material and indicate the occurrence of closely related *Cyclovirus* sequences in distinct mammalian-associated matrices within the Iberian Peninsula. However, detection in feces alone does not establish cattle as the biological host or demonstrate active infection. Complete-genome characterization and broader host and environmental surveillance are required to clarify the taxonomy, host range, and ecological significance of these viruses.

## 1. Introduction

CRESS-DNA viruses (circular Rep-encoding single-stranded DNA viruses) comprise a diverse group of small viruses characterized by circular single-stranded DNA genomes encoding a replication-associated protein (Rep). These viruses are widely distributed across a broad range of hosts and environmental niches and encompass several recognized viral families, including *Circoviridae* [1]. Members of the family *Circoviridae* are classified into two genera, *Circovirus* and *Cyclovirus*, with the latter currently comprising 90 species recognized by the International Committee on Taxonomy of Viruses (ICTV) [2]. Cycloviruses possess small circular ssDNA genomes of approximately 1.7–1.9 kb and typically contain two major open reading frames encoding the Rep and capsid (Cp) proteins [1,3].

Cycloviruses were first identified through viral metagenomic studies of human and non-human primate fecal samples, with the genus subsequently proposed in 2010 following the characterization of multiple complete circular genomes from primate stools and animal-derived food products [4].

Since their initial discovery, cycloviruses have been detected in a broad range of biological samples originating from humans and numerous animal species, including non-human primates, livestock, birds, and arthropods. Viral sequences have been identified in feces, blood, tissues, and environmental samples, suggesting that cycloviruses are widely distributed across diverse ecological niches [4]. However, the actual host range of many cycloviruses remains uncertain, particularly when viral DNA is detected in fecal material, where its presence may reflect dietary intake, environmental exposure, or passage through the gastrointestinal tract rather than active infection of the sampled host.

Although the pathogenic potential of cycloviruses remains poorly understood, *Cyclovirus* DNA has been detected in a variety of clinically relevant settings. Cycloviruses have been identified in cerebrospinal fluid from patients with suspected acute central nervous system infections and in serum and cerebrospinal fluid from patients presenting with unexplained paraplegia [5,6]. Viral sequences have also been reported in samples from individuals with febrile illness, acute flaccid paralysis, respiratory tract infections, and community-acquired pneumonia, as well as in the blood of immunocompromised patients [7,8]. Nevertheless, the detection of *Cyclovirus* DNA does not establish a causal relationship with these clinical conditions, and replication in human cells has yet to be conclusively demonstrated. Of particular interest is Human-associated *Cyclovirus* 6, currently classified within the species *Cyclovirus mmadu*, which includes strain NG12 (GQ404854), which was originally detected in the stool of a Nigerian patient with non-polio acute flaccid paralysis [9]. However, its involvement in neurological conditions remains unknown, and closely related cycloviruses have subsequently been identified in other animal-associated samples, further emphasizing the uncertainty surrounding the natural hosts and pathogenic significance of these viruses.

Despite the increasing recognition of cycloviruses across diverse animal species, knowledge of their occurrence and genetic diversity in cattle remains limited. Early studies detected *Cyclovirus*-related sequences in bovine tissues and characterized complete genomes from cattle, including strains closely related to cycloviruses identified in other livestock species, raising questions regarding their host specificity and potential for cross-species transmission [10]. However, the epidemiology of cycloviruses in cattle remains poorly understood, and information regarding their occurrence in the bovine gastrointestinal tract and fecal shedding is also scarce. Moreover, the detection of *cyclovirus* DNA in animal-derived samples does not necessarily establish the sampled species as the natural host, and the biological significance of these viruses in cattle remains unresolved. Therefore, further molecular and genomic investigations are required to clarify their occurrence, genetic diversity, and possible host associations in bovine populations. In this study, we investigated the occurrence of *cyclovirus* DNA in fecal samples collected from cattle in Portugal and performed molecular and phylogenetic characterization of the detected viral sequences. In particular, the limited molecular data available on cyclovirus-related DNA in bovine feces highlights the value of targeted surveillance studies in providing baseline information on viral occurrence and phylogenetic relationships in livestock-associated fecal material.

## 2. Materials and Methods

### 2.1. Sample Collection

A total of 100 bovine fecal samples, previously collected by convenience between July and September 2024 from 21 independent dairy farms located in 21 parishes/localities in the Viana do Castelo district, northern Portugal, were used in the present study. The participating farms were selected by convenience, without specific epidemiological or sanitary inclusion criteria.

Between 1 and 15 animals were sampled per farm (median, three animals), with each animal contributing a single fecal sample. These samples had previously been investigated for enteric protozoa [11] and were subsequently screened for *Cyclovirus*-related DNA. Thus, no additional selection of animals based on age, clinical status, or diarrheal status was performed for the present study. Fecal material had been collected from the uppermost portion of freshly deposited feces at the animals’ housing facilities to minimize contact with the surrounding environment. A new pair of disposable gloves was used for each sample, and fecal material was transferred to individual sterile, labeled containers. Rectal sampling was not performed because the samples originated from the previously conducted non-invasive survey and no animals were handled specifically for the purposes of the present study.

All animals were managed under semi-intensive production systems, and all sampled farms held officially free status for bovine brucellosis according to the available sanitary records. The sampled population comprised 78 adult cows (≥24 months and having calved at least once), 5 heifers (≥12 months and prior to first calving), and 17 calves (<12 months). At the time of sampling, diarrhea was recorded in 32 animals, including 17/78 adult cows and 15/17 calves, whereas none of the heifers presented diarrhea.

Animals were classified as diarrheic based on the consistency of the freshly voided feces observed at the time of sampling. Feces showing a clearly loose, unformed, or watery consistency were classified as diarrheic, whereas normally formed or physiologically soft feces were classified as non-diarrheic.

Each sample was collected immediately after the corresponding animal was observed defecating, allowing the fecal deposit to be reliably linked to the individual animal and its recorded clinical information. Following collection, samples were maintained at −80 °C until laboratory analysis. Information regarding the total number of cattle present on each participating farm was not consistently available and was therefore not included in the dataset.

### 2.2. DNA Extraction

DNA was extracted using the PurePrep96 automated magnetic bead-based extraction system (MolGen^®^, Veenendaal, The Netherlands), following the manufacturer’s instructions. Prior to extraction, fecal samples were diluted in phosphate-buffered saline (PBS) to prepare 10% suspensions. Then, samples were vortexed to obtain a homogeneous suspension and subsequently centrifuged at 8000× *g* for 5 min (Eppendorf, Hamburg, Germany) to remove coarse fecal debris. Following centrifugation, 200 µL of the clarified supernatant was transferred to the PurePrep96 system for automated nucleic acid extraction. The extraction procedure included lysis, nucleic acid binding to magnetic beads, sequential washing steps, and final elution. Purified DNA was recovered in a final volume of 100 µL and stored until downstream molecular analysis.

### 2.3. Molecular Detection of Circoviruses and Cycloviruses

Samples were screened for circovirus- and *Cyclovirus*-related sequences using a nested PCR assay targeting a conserved region of the replication-associated protein (*rep*) gene. The degenerate pan-Rep primers were those previously described by Li et al. (2010) [4], designed from conserved regions identified through alignment of the Rep proteins of the prototype *Cyclovirus* CyCV1-PK5006 and 12 representative *Circovirus* species. Annealing conditions were initially evaluated using both gradient and touchdown PCR approaches across a range of annealing temperatures during assay optimization. Based on these preliminary experiments, an annealing temperature of 50 °C was selected and subsequently used as a fixed annealing temperature in both rounds of the nested PCR for screening all samples.

PCR mixtures for both amplification rounds were prepared using Speedy Supreme NZYTaq 2× Green Master Mix (NZYTech, Lisbon, Portugal), according to the manufacturer’s instructions. Forward and reverse primers were each used at a final concentration of 0.4 µM. The first-round PCR was performed in a final volume of 15 µL, using 2 µL of extracted DNA as template, and created an expected 500 bp amplicon size. The nested second-round PCR was performed in a final volume of 25 µL, using 2 µL of the first-round PCR product as a template. For both amplification rounds, cycling conditions consisted of an initial denaturation at 95 °C for 5 min, followed by 40 cycles of denaturation at 95 °C for 1 min, annealing at 50 °C for 1 min, and extension at 72 °C for 1 min, followed by a final extension at 72 °C for 10 min.

The first PCR round was performed using primers CV-F1 (5′-GGIAYICCICAYYTICARGG-3′) and CV-R1 (5′-AWCCAICCRTARAARTCRTC-3′), followed by a second, nested amplification using primers CV-F2 (5′-GGIAYICCICAYYTICARGGITT-3′) and CV-R2 (5′-TGYTGYTCRTAICCRTCCCACCA-3′). Based on the optimization experiments, an annealing temperature of 50 °C was selected for both amplification rounds.

The nested PCR generated an approximately 400 bp fragment of the *rep* gene. Amplicons of the expected size were considered presumptively positive for *Circovirus*/*Cyclovirus*-related sequences and were subsequently subjected to sequence-based characterization for taxonomic identification.

Pre-amplification procedures and post-amplification handling were performed in physically separated work areas using separate equipment and consumables to minimize the risk of amplicon carry-over. A no-template control was included in each amplification run and remained negative throughout the study. The positive amplification control consisted of DNA from a gull circovirus-positive sample (GenBank accession QB005921) originating from an independent study and was genetically distinct from the Cyclovirus-related sequences detected in the bovine samples. Positive samples were confirmed by independent repeat amplification from the original DNA extract before bidirectional Sanger sequencing. No bovine sequence corresponded to the positive-control sequence.

PCR products were analyzed by agarose gel electrophoresis using a 2.5% agarose gel in SGTB buffer (Grisp, Porto, Portugal), run at 120 V for approximately 25 min, and visualized under UV illumination following staining with Xpert Green DNA Stain (Grisp, Porto, Portugal).

### 2.4. Sanger Sequencing

Amplicons of the expected size were purified and subjected to bidirectional Sanger sequencing by STAB VIDA (Caparica, Portugal). Sequencing reactions were performed using the same forward and reverse primers employed in the second round of the nested PCR, CV-F2 and CV-R2, respectively. Forward and reverse chromatograms were subsequently inspected for sequence quality, trimmed to remove low-quality regions and primer sequences, and assembled to generate consensus sequences for downstream taxonomic and phylogenetic analyses. Ambiguous nucleotide positions were manually evaluated by comparison of the corresponding peaks in both sequencing directions, and sequences with unresolved ambiguous or mixed signals were not retained for downstream analyses.

### 2.5. Sequence Analysis and Taxonomic Identification

Consensus sequences obtained from bidirectional Sanger reads were compared against the NCBI nucleotide database using online BLASTn tool (https://blast.ncbi.nlm.nih.gov/Blast.cgi, accessed on 12 October 2026) to assess their taxonomic identity and identify the closest available reference sequences. Sequence similarity was evaluated based on percentage nucleotide identity, query coverage, and alignment significance.

### 2.6. Phylogenetic Analysis

The 14 nucleotide sequences obtained in this study were analyzed together with representative *Cyclovirus* sequences retrieved from GenBank. Reference sequences were selected based on BLASTn similarity to the sequences generated in the present study and to provide broader phylogenetic representation of *Cyclovirus* lineages associated with different host species. In particular, the analysis included the closest identified reference, *Crocidura russula* feces-associated *Cyclovirus* MAVG5 (PQ576920), as well as epidemiologically and taxonomically relevant references, including Human-associated *Cyclovirus* 6 isolate NG12 (GQ404854) and Bovine-associated *Cyclovirus* 1 (HQ738634). Accession numbers and associated metadata for all reference sequences included in the analysis are provided in Supplementary Table S1.

Multiple sequence alignment was performed using MAFFT v7.526 and manually inspected to ensure positional homology. The alignment was trimmed to retain the longest homologous region represented across the dataset, resulting in a final alignment length of 377 nucleotide positions. Maximum-likelihood phylogenetic reconstruction was performed using IQ-TREE v3.1.4. ModelFinder was used to select the best-fitting nucleotide substitution model according to the Bayesian Information Criterion (BIC), resulting in the selection of the TIM2+F+I+G4 model. Branch support was assessed using 1000 ultrafast bootstrap replicates (UFBoot2). No external outgroup was included; therefore, the resulting phylogeny was treated as unrooted. The resulting tree was exported in Newick format and visualized and annotated using the Interactive Tree Of Life (iTOL) v7.6.

### 2.7. Statistical Analysis

The molecular occurrence of *Cyclovirus* was calculated as the proportion of *Cyclovirus*-related DNA-positive samples among the total number of samples analyzed. To account for the clustered sampling design, with multiple animals sampled within the same farm, 95% confidence intervals (95% CI) for molecular occurrence were estimated using cluster bootstrap resampling at the farm level with 1000 bootstrap replicates and percentile-based confidence intervals.

The relationship between *Cyclovirus* detection and diarrhea was primarily evaluated descriptively, including stratification by age category. In addition, an exploratory univariable generalized estimating equation (GEE) logistic regression model with a binomial distribution and logit link was fitted to assess the association between *Cyclovirus* detection and diarrheal status while accounting for within-farm clustering. Farm was specified as the clustering variable, and an exchangeable working correlation structure was assumed. A bias-reduced robust covariance estimator was used to account for the relatively small number of clusters. The magnitude of the association was expressed as an odds ratio (OR) with the corresponding 95% CI.

Statistical analyses were performed using Python v3.12 with statsmodels v0.14.6. Statistical significance was set at *p* < 0.05.

## 3. Results

### 3.1. Cyclovirus Detection and Molecular Occurrence

*Cyclovirus*-related DNA was detected in 14 of the 100 bovine fecal samples analyzed, corresponding to an overall molecular occurrence of 14.0% (cluster-bootstrap 95% CI: 5.6–26.5%). Positive animals originated from 7 of the 21 sampled farms (33.3%). No *circovirus*-related sequences were detected among the 100 samples analyzed.

According to age category, *Cyclovirus*-related DNA was detected in 10/78 adult cattle (12.8%; 95% CI: 6.3–22.3%), 1/5 heifers (20.0%; 95% CI: 0.5–71.6%), and 3/17 calves (17.6%; 95% CI: 3.8–43.4%). Given the limited number of animals in some age categories, particularly heifers, these subgroup proportions should be considered descriptive. The number of animals tested and the number of Cyclovirus-related DNA-positive animals for each sampled farm are provided in Supplementary Table S2.

### 3.2. Sequence Characterization

Bidirectional Sanger sequencing generated 14 *Cyclovirus*-related consensus sequences suitable for molecular characterization and phylogenetic analysis. Following quality-based trimming of the terminal regions of the chromatograms, the resulting high-quality sequences ranged from 230 to 377 bp in length. All sequences corresponded to the same homologous region of the *rep* gene targeted by the nested PCR assay.

Comparison with the *Crocidura russula* feces-associated *Cyclovirus* MAVG5 reference sequence (PQ576920) revealed high nucleotide similarity, ranging from 94.8% to 100% across the analyzed homologous region. Five sequences showed 100% nucleotide identity to the corresponding region of PQ576920, while the remaining sequences displayed identities ranging from 94.8% to 99.7%.

### 3.3. Phylogenetic Analysis

Maximum-likelihood phylogenetic analysis placed all 14 bovine-derived sequences within a closely related *Cyclovirus* lineage together with the *Crocidura russula* feces-associated *Cyclovirus* MAVG5 (PQ576920). The clade comprising the bovine sequences and PQ576920 was supported by a bootstrap value of 90.9%.

**Figure 1:**
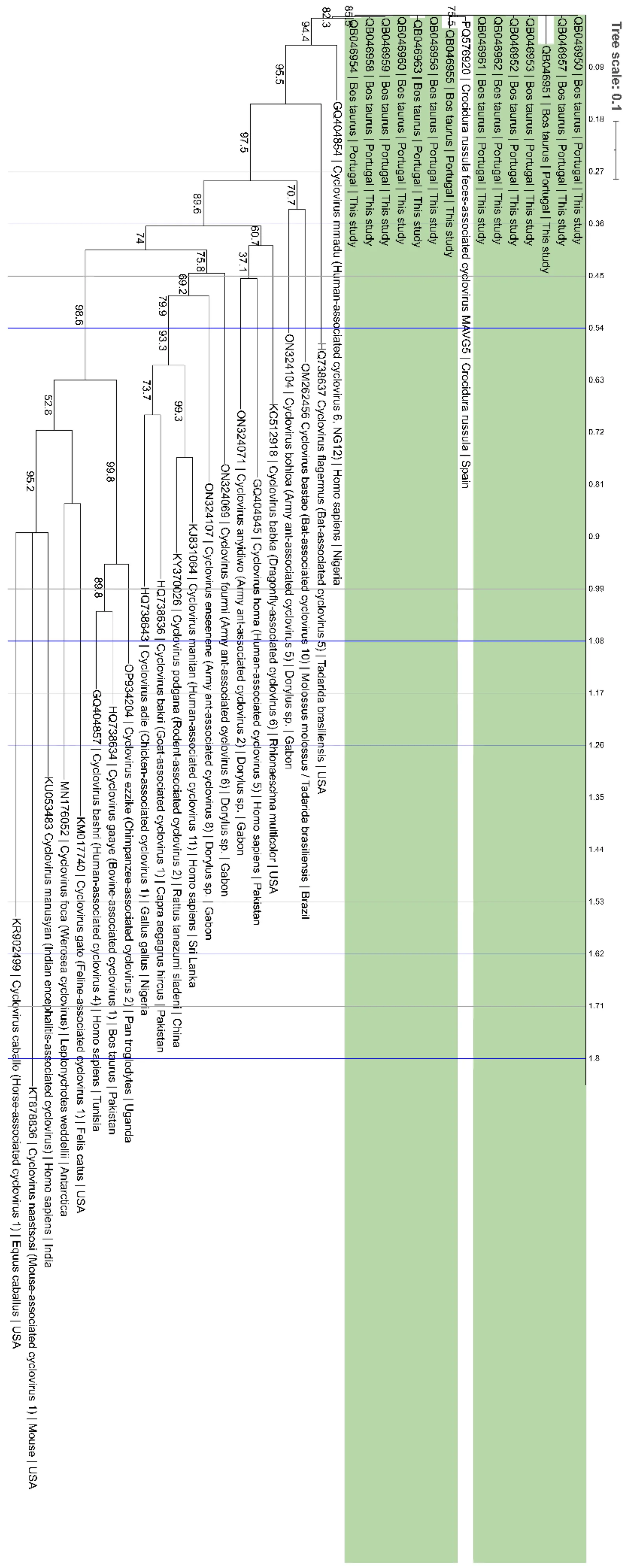
Maximum-likelihood phylogenetic tree based on partial *rep* gene sequences of *Cyclovirus*. Phylogenetic reconstruction was performed using the TIM2+F+I+G4 nucleotide substitution model, selected as the best-fitting model by ModelFinder in IQ-TREE according to the Bayesian Information Criterion (BIC). Node support values represent bootstrap percentages based on 1000 ultrafast bootstrap replicates (UFBoot2). Reference sequence labels include the GenBank accession number, virus/species designation, host species, and country of origin. Sequences obtained in the present study are highlighted in green. The scale bar represents 0.1 nucleotide substitutions per site. The accession numbers of the reference sequences included in the phylogenetic analysis are provided in Supplementary Table S1.

This group clustered with Human-associated *Cyclovirus* 6 isolate NG12 (GQ404854; *Cyclovirus mmadu*), with 99.4% bootstrap support for the broader lineage. In contrast, Bovine-associated *Cyclovirus* 1 (HQ738634; *Cyclovirus gaaye*) occupied a distinct and comparatively distant phylogenetic lineage. Internal relationships among the highly similar bovine-derived sequences generally showed lower bootstrap support. This pattern is illustrated in Figure 1.

### 3.4. Association Between Cyclovirus Detection and Diarrhea

Among the 32 animals presenting diarrhea, 3 (9.4%) were positive for Cyclovirus-related DNA, compared with 11 of the 68 non-diarrheic animals (16.2%). The distribution differed substantially according to age category. Among adult cows, none of the 17 diarrheic animals was positive, compared with 10/61 non-diarrheic animals. Among calves, 3/15 diarrheic animals were positive, whereas neither of the two non-diarrheic calves was positive. None of the five heifers presented diarrhea, and the single Cyclovirus-positive heifer was non-diarrheic.

In an exploratory univariable GEE analysis accounting for within-farm clustering, no statistically significant association was observed between Cyclovirus detection and diarrhea (OR = 0.76, 95% CI: 0.31–1.86; *p* = 0.549). Given the small number of positive animals and the marked relationship between age category and diarrheal status, this estimate should be interpreted cautiously and may be affected by confounding by age.

Given the limited number of *Cyclovirus*-positive animals, the small number of positive animals presenting diarrhea, and the sparse distribution of diarrhea across age categories, an age-adjusted multivariable model was not fitted because it was considered unlikely to provide a sufficiently stable estimate. Accordingly, the univariable GEE analysis was considered exploratory and interpreted cautiously in view of potential confounding by age.

Individual molecular and epidemiological characteristics of the 14 *Cyclovirus*-related DNA positive samples, including GenBank accession numbers, are provided in Supplementary Table S3.

## 4. Discussion

In the present study, *Cyclovirus* DNA was detected in 14 of the 100 bovine fecal samples analyzed. Sequence characterization of the partial *rep* gene revealed high nucleotide similarity to the *Crocidura russula* feces-associated *Cyclovirus* strain MAVG5 (PQ576920), with pairwise identities ranging from 94.8% to 100% and 100% query coverage in the BLAST comparisons. These findings indicate the fecal presence of closely related *Cyclovirus* sequences among cattle from northern Portugal.

Although MAVG5 was originally recovered from fecal samples of *Crocidura russula* in Spain, complete-genome analysis demonstrated that this virus is closely related to Human-associated *Cyclovirus* 6 [12]. Specifically, MAVG5 showed 97.9% nucleotide identity with 99% query coverage to the Human-associated *Cyclovirus* 6 strain RI46/ITA (MZ201304), while genome-wide pairwise comparison revealed 89.2% nucleotide identity. According to current ICTV criteria, members of the genus *Cyclovirus* are assigned to different species when their complete genomes share less than 80% genome-wide nucleotide sequence identity [2]. Therefore, MAVG5 and Human-associated *Cyclovirus* 6 are considered variants belonging to the same species rather than distinct species [12].

The detection of *Cyclovirus*-related DNA in bovine feces in the present study is relevant in the context of the still limited knowledge regarding *Cyclovirus* occurrence in farm animals. Despite the growing number of cycloviruses detected in diverse biological matrices, including human feces and tissues from cattle, goats, and birds, information regarding their occurrence and prevalence in fecal samples from livestock remains scarce [13]. Consequently, the epidemiological significance, host range, and potential circulation of cycloviruses within livestock populations are still poorly understood.

This knowledge gap is particularly evident in cattle. Although diverse CRESS-DNA viruses have previously been identified in bovine fecal samples, bovine-associated cycloviruses have primarily been detected in tissue samples [4], whereas their occurrence and prevalence in bovine feces remain largely unexplored. The detection frequency observed in the present study (14%, 14/100) provides new evidence that *Cyclovirus*-related DNA may be relatively common in the fecal material of cattle within the studied population. Direct comparison with previous bovine studies is difficult, since most investigations of bovine feces have focused on other CRESS-DNA viruses rather than members of the genus *Cyclovirus* [3]. For example, Bo-Circo-like viruses have been detected in fecal samples from cattle, including an initial detection rate of 5.3% (4/75) among diarrheic animals and, subsequently, a larger survey reporting viral DNA in 23.6% (205/868) of cattle examined across China [3,14]. However, these viruses are genetically distinct from *Cyclovirus* and are currently associated with the Kirkoviridae lineage. Consequently, these prevalence estimates cannot be directly compared with the 14% detection frequency observed here, but they illustrate the considerable diversity of CRESS-DNA viruses detectable in the bovine gastrointestinal environment [15].

Although *Cyclovirus*-related DNA was detected less frequently among diarrheic animals than among non-diarrheic animals (9.4% vs. 16.2%), the cluster-adjusted analysis did not identify a statistically significant association with diarrhea. However, the limited number of positive animals and the resulting wide confidence interval restrict the precision of this estimate; therefore, the absence of statistical significance should not be interpreted as evidence that an association is absent.

Importantly, detection of *Cyclovirus* DNA in feces should not, by itself, be interpreted as evidence of active infection of cattle [16]. Fecal samples represent complex biological matrices containing material derived not only from the host but also from dietary, environmental, microbial, and potentially parasitic sources [9,16].

The same fecal samples had previously been screened for *Giardia duodenalis*, which was detected in six animals [11]. Only one of the 14 *Cyclovirus*-related DNA-positive samples was also positive for *G. duodenalis*, indicating limited overlap between the two detections [11]. While this observation does not allow for conclusions to be drawn regarding the origin of the detected *Cyclovirus*-related DNA, the low level of co-detection suggests that its presence was not consistently associated with *G. duodenalis* positivity in the sampled animals. Overall, co-detection of *Giardia duodenalis* and cyclovirus-related DNA was observed in only one of the 100 bovine fecal samples analyzed.

Host assignment is particularly challenging for CRESS-DNA viruses identified exclusively through molecular approaches, and the detection of a viral sequence in the gastrointestinal tract does not necessarily identify the organism in which viral replication occurred. Therefore, the present findings demonstrate the presence of *Cyclovirus*-related DNA in bovine feces, but further evidence would be required to establish cattle as a biological host of these viruses [15,16]. In addition, although the degenerate *rep*-targeting assay used in this study was designed for broad detection of both *Cyclovirus*- and circovirus-related sequences, no circovirus-related sequences were identified. This finding should be interpreted as non-detection under the employed screening conditions rather than evidence of their complete absence from the sampled population.

The uncertainty surrounding host assignment is particularly relevant given the close genetic relationship between the sequences detected here and MAVG5, which was originally identified in feces of the insectivorous mammal *Crocidura russula* [12]. The occurrence of highly similar *Cyclovirus* sequences in fecal samples from phylogenetically distant mammalian species raises questions regarding their actual host range and ecological origin. Similar observations have previously been reported for cycloviruses, including closely related viral genomes recovered from different farm animal species. For instance, a *Cyclovirus* recovered from goat tissue was found to be nearly identical to a bovine-associated *Cyclovirus*, supporting the possibility that genetically related cycloviruses may occur across different animal species. However, the identification of genetically related viral sequences in samples from different animal species does not necessarily establish these animals as biological hosts or provide evidence of cross-species transmission.

The geographical context of the present findings is also noteworthy. MAVG5 was identified in *C. russula* feces collected in Spain, whereas the closely related sequences described here were detected in cattle sampled in northern Portugal [12]. Recent metagenomic analyses of yak (*Bos grunniens*) feces have likewise revealed a diverse assemblage of CRESS-DNA viruses, including members of the family *Circoviridae*, further illustrating the broad occurrence of these viruses in ruminant-associated fecal viromes [17].

Although the available data are insufficient to infer transmission between wildlife and livestock, the detection of genetically similar sequences in neighboring countries and distinct mammalian-associated matrices suggests that this *Cyclovirus* lineage may have a broader ecological distribution in the Iberian Peninsula than currently recognized. Expanded surveillance encompassing livestock, sympatric wildlife, environmental samples, and other potential hosts would be required to clarify its distribution and ecological associations.

Several limitations of the present study should be acknowledged. First, the relatively limited sample size and the restricted geographical origin of the samples, which were obtained from cattle farms in northern Portugal, limit the extent to which the observed 14% detection frequency can be extrapolated to the wider bovine population. In addition, the cross-sectional nature of the study does not allow for assessment of temporal patterns, persistence, or intermittent detection of *Cyclovirus* DNA. Only fecal material was examined, and no paired blood or tissue samples were available to investigate systemic presence or provide further evidence supporting cattle as the biological host. Furthermore, viral load was not quantified, and no attempts were made to demonstrate viral replication or recover infectious virus. Moreover, viral isolation was beyond the scope of this molecular investigation and is further complicated by the absence of established cell culture systems for members of the genus *Cyclovirus*, whose definitive hosts and replication biology remain poorly understood. Additionally, the absence of extraction blanks and internal amplification controls limits the ability to exclude contamination during DNA extraction and PCR inhibition in fecal samples, respectively. Although a genetically distinct circovirus-positive control was included, the analytical sensitivity of the assay for the specific cyclovirus lineage detected was not determined. The relatively small number of *Cyclovirus*-related DNA positive animals and positive farms also limited the precision of association estimates, as reflected by the wide confidence intervals.

Therefore, the present results should primarily be interpreted as evidence of the occurrence of *Cyclovirus*-related DNA in bovine fecal samples, rather than evidence of active infection or disease in cattle.

Finally, the present study is limited by the characterization of a partial region of the *rep* gene. Although this region allowed the detected sequences to be compared with previously described cycloviruses and supported their phylogenetic placement, partial *rep* sequences are insufficient for definitive species-level taxonomic assignment under current ICTV criteria [2], which rely on genome-wide nucleotide identity. Recovery of complete circular genomes through approaches such as rolling-circle amplification followed by inverse PCR or metagenomic sequencing would therefore be important to determine the precise taxonomic position of the bovine-associated sequences, characterize their genomic organization, and establish their relationship with MAVG5 and Human-associated *Cyclovirus* 6.

## 5. Conclusions

This study identified *cyclovirus*-related DNA in 14% of bovine fecal samples collected in northern Portugal and revealed high genetic similarity between the partial *rep* sequences obtained and the *Crocidura russula* feces-associated *cyclovirus* MAVG5. These findings expand current knowledge on the occurrence of *cyclovirus*-related sequences in livestock-associated fecal material and highlight the detection of genetically similar sequences in fecal samples from distinct mammalian species within the Iberian Peninsula. However, detection in feces alone does not establish cattle as the biological host or demonstrate active infection, replication, or pathogenicity. Recovery of complete viral genomes, together with broader surveillance including additional animal species, tissues, blood, and environmental samples, will be necessary to clarify the taxonomy, host associations, epidemiology and ecological significance of these viruses.

## Data Availability

The partial *rep* gene sequences generated in this study have been deposited in GenBank under accession numbers QB046950–QB046963. Additional data supporting the findings of this study are available within the article and its Supplementary Materials. The phylogenetic alignment and tree files, as well as other data, are available from the corresponding author upon reasonable request.

## Supplementary Materials

The following supporting information can be downloaded at https://www.mdpi.com/article/10.3390/microorganisms14102374/s1, Table S1. Reference *Cyclovirus* sequences included in the phylogenetic analysis, with corresponding GenBank accession numbers, virus or species designations, host or sample sources, and geographical origins. Table S2. Distribution of bovine fecal samples tested and samples positive for cyclovirus-related DNA across the 21 sampling locations in the Viana do Castelo region, northern Portugal. Table S3. Molecular and epidemiological characteristics of bovine fecal samples positive for cyclovirus-related DNA, including nucleotide sequence identity, query coverage, E-values, and bit scores obtained from BLASTn comparisons with *Crocidura russula* feces-associated cyclovirus MAVG5 (GenBank accession PQ576920.1).
