# Supplementary material for: Cyclovirus-Related DNA in Bovine Feces from Northern Portugal: Molecular Detection and Phylogenetic Characterization
Source: Microorganisms. 2026 Oct 16;14(10):2374. doi: 10.3390/microorganisms14102374 (PMC13707405; doi:10.3390/microorganisms14102374)
Supplement: Supplementary file 1 [file microorganisms-14-02374-s001.zip › microorganisms-4620448-supplementary.pdf]

Table S1. Reference *Cyclovirus* sequences included in the phylogenetic analysis.

GenBank accession	Virus/species designation	Host/source	Country
**PQ576920**	*Crocidura russula* feces-associated *Cyclovirus* MAVG5	*Crocidura russula* feces	Spain
**GQ404854**	*Cyclovirus mmadu* (Human-associated cyclovirus 6; NG12)	*Homo sapiens*	Nigeria
**HQ738637**	*Cyclovirus flagermus* (Bat-associated cyclovirus 5)	*Tadarida brasiliensis*	USA
**KM382270**	*Cyclovirus bastao* (Bat-associated cyclovirus 10)	*Molossus molossus / Tadarida brasiliensis*	Brazil
**ON324104**	*Cyclovirus bohloa* (army ant-associated cyclovirus 5)	*Dorylus* sp.	Gabon
**KC512918**	*Cyclovirus babka* (Dragonfly-associated cyclovirus 6)	*Aeshna multicolor*	USA
**GQ404845**	*Cyclovirus homa* (Human-associated cyclovirus 5)	*Homo sapiens*	Pakistan
**ON324071**	*Cyclovirus anyidiwo* (army ant-associated cyclovirus 2)	*Dorylus* sp.	Gabon
**ON324069**	*Cyclovirus fourmi* (army ant-associated cyclovirus 6)	*Dorylus* sp.	Gabon
**ON324107**	*Cyclovirus enseenene* (army ant-associated cyclovirus 8)	*Dorylus* sp.	Gabon
**KJ831064**	*Cyclovirus manitan* (Human-associated cyclovirus 11)	*Homo sapiens*	Sri Lanka
**KY370026**	*Cyclovirus podgana* (Rodent-associated cyclovirus 2)	*Rattus tanezumi sladeni*	China
**HQ738636**	*Cyclovirus bakri* (Goat-associated cyclovirus 1)	*Capra aegagrus hircus*	Pakistan
**HQ738643**	*Cyclovirus adie* (Chicken-associated cyclovirus 1)	*Gallus gallus*	Nigeria
**OP934204**	*Cyclovirus ezzike* (Chimpanzee-associated cyclovirus 2)	*Pan troglodytes*	Uganda
**HQ738634**	*Cyclovirus gaaye* (Bovine-associated cyclovirus 1)	*Bos taurus*	Pakistan

**GQ404857**	*Cyclovirus bashri* (Human-associated cyclovirus 4)	*Homo sapiens*	Tunisia
**KM017740**	*Cyclovirus gato* (Feline-associated cyclovirus 1)	*Felis catus*	USA
**MN176052**	*Cyclovirus foca*	*Leptonychotes weddellii*	Antarctica
**KU053483**	*Cyclovirus manusyan* (Human-associated cyclovirus 12)	*Homo sapiens*	India
**KT878836**	*Cyclovirus naastsosi* (Mouse-associated cyclovirus 1)	Mouse	USA
**KR902499**	*Cyclovirus caballo* (Horse-associated cyclovirus 1)	*Equus caballus*	USA

Table S2: Number of bovine fecal samples tested and Cyclovirus-related DNA-positive samples according to sampling location.

Sampling location	Animals tested, n	Positive animals, n
**Carreço**	13	0
**Cerveira (Campos)**	2	2
**Vila Franca**	12	2
**Meadela**	8	1
**Deão**	3	3
**Areosa**	1	0
**Mozelos**	1	0
**Outeiro**	2	0
**Vila Fria**	11	2
**Subportela**	15	0
**Darque**	3	0

**Afife**	3	0
**Deocriste**	1	0
**Alvarães**	3	0
**Agros**	3	2
**Perre**	2	0
**Torre e Vila Mou**	1	0
**Santa Marta de Portuzelo**	3	2
**Carvoeiro**	9	0
**Castelo de Neiva**	2	0
**Samonde**	2	0
**Total**	**100**	**14**

Table S3. Molecular and epidemiological characteristics of cyclovirus-related DNA-positive bovine fecal samples and sequence comparisons with Crocidura russula feces-associated cyclovirus MAVG5 (PQ576920.1)

**Sample ID**	**Collection date**	**Sampling location**	**Age category**	**Diarrhea**	**Nucleotide identity (%)**	**Query coverage (%)**	**Reference accession**	**Sequence length (bp)**	**GenBank accession**	**E-value**	**Bit score**
**14**	09/07/2024	Cerveira (Campos)	Adult	No	100.0	100.0	PQ576920.1	356	QB046950	2.02×10^{-179}	641
**15**	09/07/2024	Cerveira (Campos)	Adult	No	94.8	100.0	PQ576920.1	230	QB046951	1.40×10^{-89}	342
**21**	12/07/2024	Deão	Adult	No	99.7	100.0	PQ576920.1	377	QB046952	0.0	675
**22**	12/07/2024	Deão	Adult	No	99.7	100.0	PQ576920.1	366	QB046953	0.0	654
**23**	12/07/2024	Deão	Adult	No	99.4	100.0	PQ576920.1	362	QB046954	2.06×10^{-179}	641

**38**	31/07/2024	Vila Franca	Adult	No	97.6	99.4	PQ576920.1	341	QB046955	5.51×10^{-160}	577
**40**	31/07/2024	Vila Franca	Adult	No	100.0	100.0	PQ576920.1	363	QB046956	0.0	654
**52**	06/08/2024	Meadela	Calf	Yes	99.7	100.0	PQ576920.1	363	QB046957	0.0	649
**53**	08/08/2024	Agros	Adult	No	99.7	100.0	PQ576920.1	351	QB046958	5.57×10^{-175}	627
**54**	08/08/2024	Agros	Adult	No	99.7	100.0	PQ576920.1	373	QB046959	0.0	667
**60**	27/08/2024	Vila Fria	Calf	Yes	100.0	100.0	PQ576920.1	365	QB046960	0.0	658
**67**	28/08/2024	Santa Marta de Portuzelo	Calf	Yes	100.0	99.7	PQ576920.1	337	QB046961	2.49×10^{-168}	604
**68**	28/08/2024	Santa Marta de Portuzelo	Heifer	No	100.0	100.0	PQ576920.1	338	QB046962	1.93×10^{-169}	608
**69**	03/09/2024	Vila Fria	Adult	No	99.7	98.6	PQ576920.1	348	QB046963	1.55×10^{-170}	612
